# Supplementary material for: Molecular Cloning, Expression Analysis, and Functional Analysis of Nine IbSWEETs in Ipomoea batatas (L.) Lam
Source: Int J Mol Sci. 2023 Nov 22;24(23):16615. doi: 10.3390/ijms242316615 (PMC10706379; doi:10.3390/ijms242316615)
Supplement: Supplementary file 1 [file ijms-24-16615-s001.zip › ijms-2674942-supplementary.pdf]

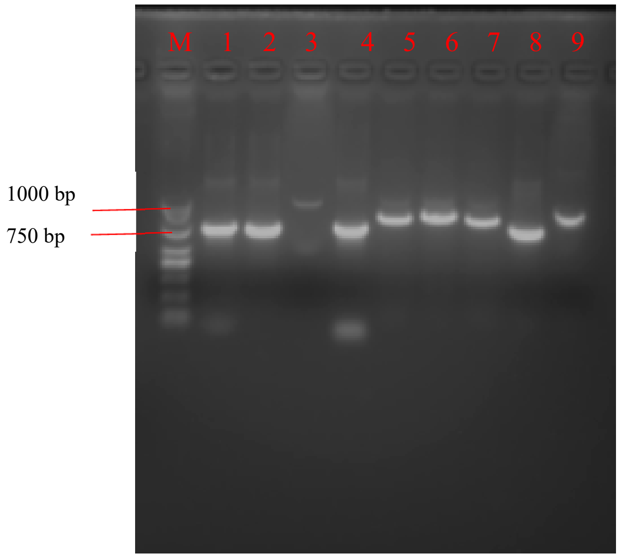

Figure S1. Agarose gel diagram of *SWEET* genes PCR amplification results. M: marker 2000bp; 1-9 were the PCR results of *IbSWEET1a, 1b, 2, 7, 10a, 10c, 12, 15,* and *17,* respectively.

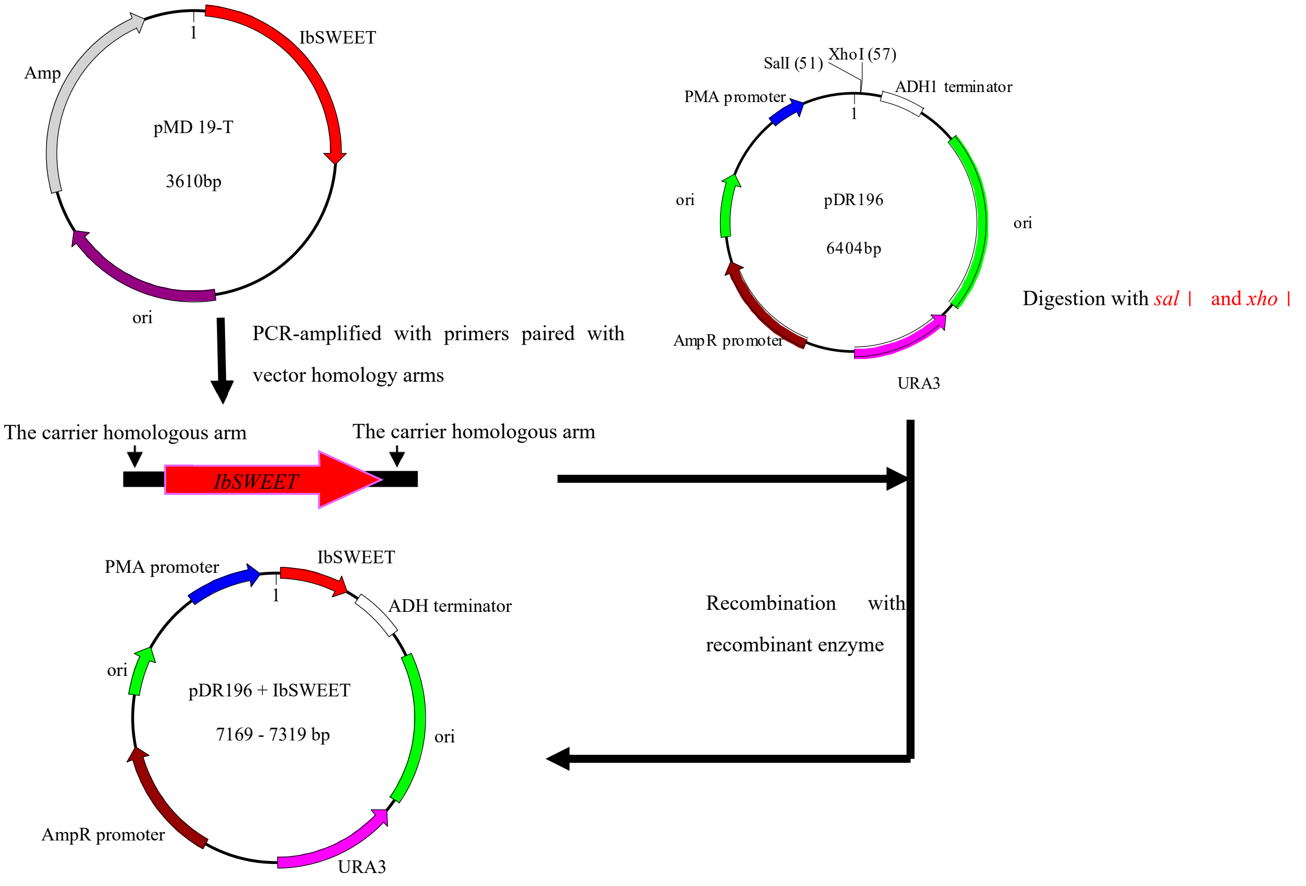

Figure S2. Construction of *IbSWEETs* expression vector.
